# MET and Small-Cell Lung Cancer

**DOI:** 10.3390/cancers6042100

**Published:** 2014-10-13

**Authors:** Francesco Gelsomino, Giulio Rossi, Marcello Tiseo

**Affiliations:** 1 Medical Oncology Unit 1, Medical Oncology Department, Fondazione IRCCS Istituto Nazionale Tumori, Via G. Venezian 1, 20133 Milano, Italy; 2 Operative Unit of Pathology, Azienda Ospedaliero-Universitaria Policlinico, Via del Pozzo 71, 41124 Modena, Italy; E-Mail: giurossi68@gmail.com; 3 Medical Oncology Unit, Azienda Ospedaliero-Universitaria, Viale A. Gramsci 14, 43126 Parma, Italy; E-Mail: mtiseo@ao.pr.it

**Keywords:** c-MET, small-cell lung cancer, mutations, c-MET signaling pathway

## Abstract

Small-cell lung cancer (SCLC) is one of the most aggressive lung tumors. The majority of patients with SCLC are diagnosed at an advanced stage. This tumor type is highly sensitive to chemo-radiation treatment, with very high response rates, but invariably relapses. At this time, treatment options are still limited and the prognosis of these patients is poor. A better knowledge of the molecular biology of SCLC allowed us to identify potential druggable targets. Among these, the MET/HGF axis seems to be one of the most aberrant signaling pathways involved in SCLC invasiveness and progression. In this review, we describe briefly all recent literature on the different molecular profiling in SCLC; in particular, we discuss the specific alterations involving *c-MET* gene and their implications as a potential target in SCLC.

## 1. Introduction

Lung cancer remains the most common cause of cancer-related death worldwide [1]. Small-cell lung cancer (SCLC) accounts for 12%–15% of all lung cancers. It most often occurs in smokers and has a very aggressive behavior due to its high proliferative index; in fact, about 60%–70% of SCLC patients have a metastatic disease at the time of diagnosis. Despite the high sensitivity to chemo- and radiation therapy, the long-term outcome of the majority of SCLC patients remains poor due to early relapse and acquired resistance [2].

Patients with limited-stage (LD) SCLC are candidates for a combination treatment of chemo- and radio-therapy and for consecutive prophylactic cranial irradiation (PCI) in case of tumor response. These patients achieve a response rate (RR) over 60%–70%, with a median overall survival (mOS) of 16–24 months and a two-year survival rate of 25% [3,4,5]. Patients with extensive-stage (ED) are candidates for chemotherapy, producing RRs of 50%–60%, and PCI in cases of tumor response; for them, mOS is 8–13 months and the two-year survival rate is dismal (about 5%) [6].

In the last decades, little progress has been made in the treatment of SCLC and the prognosis of these patients remains poor; therefore, novel therapies are urgently required.

The knowledge of new potential therapeutic targets might be able to enhance the efficacy of the standard treatments by concurrent administration or as salvage after failure of standard therapies.

## 2. Molecular Profiling of Small-Cell Lung Cancer (SCLC)

As happened for non-small-cell lung cancer (NSCLC), the detection of the genomic aberrations underlying SCLC invasiveness and progression is crucial for developing new therapeutic agents and improving patients’ outcome. The main genomic aberrations in SCLC are summarized in Table 1.

Inactivating mutations in *TP53* and *Rb1* genes are frequent, reaching a very high prevalence (up to 90%); conversely, *c-MYC* amplification, activating mutations in *EGFR*, *KRAS*, *PIK3CA* genes, c-KIT overexpression and mutation/loss of PTEN are rare events [7,8,9,10,11].

Recently, Wakuda *et al.* [12] assessed the prevalence of several genomic alterations in 60 SCLCs by using a multiplexed tumor genotyping platform. Thirteen genomic alterations were detected in 15% of the cases and *PIK3CA* was identified as one of the prevalent aberrant genes.

Other studies have been conducted using different high-throughput tumor genotyping platforms. In one study, 51 resected SCLC samples were analyzed and genetic alterations in PIK3CA pathway and *PIK3CA* mutations were detected in 36% and 6% of all cases, respectively [13].

In addition, two independent genomic analyses identified specific driver alterations in SCLC. In the first study [14], 99 SCLC specimens were analyzed and inactivating mutations/loss in *TP53* and *Rb1* genes were observed in almost all cases. Mutations in the *PTEN* gene, responsible for stimulating the activation of PI3K pathway, were identified in 10% of cases. No mutation in the *PIK3CA* gene was detected. Other gene alterations included: inactivating mutations in *CREBBP* and *EP300* genes, *MYC* and *FGFR1* genes amplifications. In particular, *PTEN* mutations and *FGFR1* amplifications may represent potential druggable genome alterations. Other studies confirmed that, although rare events, *FGFR1* gene alterations predict sensitivity to FGFR inhibition both *in vitro* and in xenograft models [15,16].

In the second study [17], 80 human SCLC, including also 40 SCLC cell lines, were analyzed by applying next-generation sequencing technologies. Twenty-two mutated genes were identified. *TP53* and *Rb1* genes were frequently mutated. Mutations involving other genes, not known previously in SCLC, were detected. Furthermore, *SOX2* amplification/overexpression was observed in 27% and *RLF–MYCL1* gene fusions in 9% of SCLC samples. These alterations may be considered as oncogenic-drivers and consequently as two possible druggable targets.

**Table 1:** Genomic aberrations in SCLC.

Author	no. of Samples	Test(s)	no. (%) of Genomic Alterations	Type of Alterations
Shibata [9]	15 tumors	Direct sequencing	2 (13%)	*PIK3CA* mut
	13 cell lines		3 (23%)	
Yokomizo [10]	10 tumors	DHPLC Direct sequencing	1 (10%)	*PTEN/MMAC1* mut
	34 cell lines		6 (18%)	
Tatematsu [11]	122 tumors	Direct sequencing FISH	5 (4%)	*EGFR* mut
			4 (3%)	*EGFR* ampl
Wakuda [12]	60 tumors	Pyrosequencing	13 in 9 cases (15%)	4 *PIK3CA* ampl, 1 *PIK3CA* mut, 1 *EGFR* mut + *PIK3CA* mut, 1 *KRAS* mut, 1 *AKT1* mut + *PIK3CA* ampl, 1 *PIK3CA* mut + *MET* ampl + *PIK3CA* ampl
Umemura [13]	51 tumors	Whole-exome sequencing, copy-number analysis	18 (36%)	*PIK3CA* pathway
			3 (6%)	*PIK3CA* mut
Peifer [14]	97 tumors 2 cell lines	SNP; exome-, transcriptome- and genome-sequencing	29 (100%)	*TP53/Rb1* mut and loss
			18 (18%)	*CREBBP/EP300* mut
			10 (16%)	*MYC* ampl
			9 (10%)	*SLIT2* mut
			3 (10%)	*PTEN* mut
			3 (10%)	*EPHA7* mut
			3 (10%)	*MLL* mut
			3 (6%)	*FGFR1* ampl
Rudin [17]	40 tumors 40 cell lines	Exome sequencing, RNA-sequencing, whole-genome sequencing, RT-PCR, FISH and IHC	33 (78%)	*TP53* mut
			14 (33%)	*Rb1* mut
			15 (27%)	*SOX2* ampl
			5 (9%)	*RLF-MYCL1* fusion gene
	
Ma [18]	32 tumors	Sequencing	4 (12%)	*JM* mut, *Sema* mut, pre-*JM* intron 13 mut
	10 cell lines		3 (30%)	*JM* mut, alternative transcript involving exon 10
de Aguirre [19]	44 tumors	Direct sequencing	3 (8%)	*JM* mut, *Sema* mut
Voortman [20]	46 tumors	Sequencing	3 (6.5%)	*JM* mut
	13 cell lines		3 (25%)	
Bordi [21]	113 tumors	Direct sequencing	5 (4.4%)	*JM* mut, *TK* mut

PCR, polymerase chain reaction; mut, mutation; DHPLC, denaturing high-performance liquid chromatography; RT-PCR, reverse transcriptase polymerase chain reaction; FISH, fluorescent *in situ* hybridization; ampl, amplification; SNP, single nucleotide polymorphism; IHC, immunohistochemistry.

By using an integrative proteomic and transcriptomic analysis and after a comparison between 34 SCLC and 74 NSCLC cell lines, other researchers investigated proteomic profiling with the aim to identify dysregulated pathways in SCLC [22]. In SCLC, significantly higher levels of E2F1-regulated factors, thymidylate synthase (TS), DNA repair and apoptosis proteins were detected. PARP1, a DNA repair protein and E2F1 co-activator, was significantly higher at the mRNA and protein levels both in SCLC cells lines and tumors than in NSCLCs. *In vitro*, SCLC cells were sensitive to PARP inhibitors both alone and in combination with chemotherapy; moreover, PARP1 levels correlated with PARP inhibitor sensitivity, supporting it as potential target in SCLCs.

Data from the literature show that also genomic aberrations in MET/HGF axis and its downstream modulators seem to be involved in SCLC invasiveness and progression. In contrast to the strong scientific evidence on NSCLC, few data are available on SCLC and MET to date. According to these concepts, in conjunction with insufficient treatment options in SCLC and its poor prognosis, we decided to discuss the role of the aberrant MET/HGF pathway in SCLC for the possible implications in clinical research and drug development.

## 3. MET: Structure, Function and Aberrant Signaling in Tumor

The *MET* proto-oncogene is located on chromosome 7q21-31 and encodes the receptor tyrosine kinase MET. It was first identified in a case of human osteosarcoma tumor cells exposed to *N*-methyl-N0-nitroso-guanidine which led to a fusion protein between the translocated promoter region (TPR) on chromosome 1 and MET domain on chromosome 7 [23]. Its principal ligand is hepatocyte growth factor (HGF), also called scatter factor (SF).

**Figure 1:**
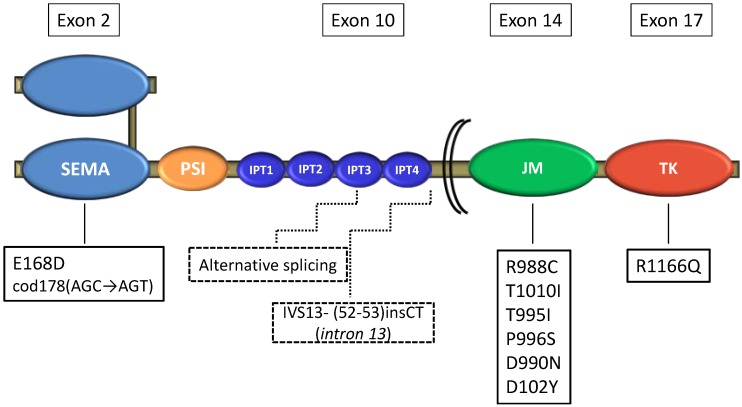
Structure of c-MET receptor and identified MET gene mutations in SCLC. SEMA, Semaphorin domain; PSI, plexins-semaphorin-integrin domain; IPT1-4, four immunoglobulin plexins transcription domains; JM, juxtamembrane domain; TK, tyrosine kinase domain.

The c-MET receptor is a disulfide-bound heterodimer expressed primarily in different epithelial cells and comprising an extracellular α-subunit and a transmembrane β-subunit. The extracellular portion includes: the Sema domain, responsible for its bond with the β-chain, PSI (also present in the plexins, semaphorins and integrins) domain and four Ig-like repeated domains (IPT), connecting the PSI domain to the transmembrane helix. The intracellular domain includes: a juxtamembrane (JM) sequence which downregulates the MET kinase activity through the phosphorylation of Ser975 and Tyr1003 residues; a catalytic region which positively modulates the kinase activity and, finally, a carboxy-terminal multifunctional docking site, responsible for the recruitment of many intracellular transducers and adaptors [24] (Figure 1).

HGF/SF is a protein belonging to the serine protease family and produced in cells of mesenchymal origin, such as fibroblasts and smooth muscle cells. It is secreted as a single chain, biologically inactive, and converted into its mature form by extracellular proteases, after a cleavage process. Its biologically active form consists of a disulfide-bond heterodimer containing an α-chain and a β-chain. The first contains an amino-terminal hairpin loop (HL) domain followed by four peculiar domains, known as kringle domains; the latter contains a serine proteases homology (SPH) domain that lacks proteolytic activity [24]. HGF/SF contains two MET receptor binding sites with different affinities and roles. The high affinity site is located into the α-chain and recognizes the IPT3 and IPT4 domains of MET receptor also independently of HGF maturation; conversely, the low-affinity site, necessary for MET dimerization, is located into the β-chain and recognizes the Sema domain only when HGF is active [25,26].

Upon binding HGF/SF, MET receptor dimerizes resulting in the activation of tyrosine kinase domain through a process of trans-phosphorylation of the two tyrosine residues Y1234 and Y1235 and, in turn, of two docking tyrosines (Y1349 and Y1356). This process leads to the recruitment of several adaptor proteins (Gab1 and Grb2), which in turn activate different intracellular signaling pathways (RAS-RAF-MEK-ERK and PI3K-AKT cascades, the nuclear factor-kB complex and STAT3), responsible for driving proliferation, cell survival, morphogenesis, cell scattering, migration and invasiveness [27,28,29,30,31].

In normal tissue, the interaction between c-MET receptor and HGF is crucial for embryonic development and organ regeneration.

During embryogenesis a normal function of HGF/MET axis plays an important role in liver, placenta and muscle formation [32,33,34], as well as nervous system [35,36,37].

In the adults, the MET-driven signaling mechanisms are involved in liver, kidney and epidermis regeneration, after exposure to acute and chronic injuries [38,39,40,41].

In several tumors, MET signaling pathway is aberrantly activated and represents one of the most important mechanisms of progression and invasiveness [42,43]. It is considered a late event able to confer on tumor cells a more aggressive phenotype, a biological phenomenon known as oncogene expedience [44]. Furthermore, aberrant MET signaling activation has been identified as a prognostic factor of poor outcome in different solid tumors [45,46,47] and also in lung cancers [48,49,50,51,52,53].

The MET pathway can be aberrantly activated as a consequence of HGF or HGFR transcriptional upregulation, gene amplification and, rarely, as the final event of a MET gene mutation [54,55,56]. Other signaling co-receptors, such as EGFR, KRAS, plexins B, integrin and CD44v6, can cross-talk with the MET receptor, even in a HGF-independent manner, providing an alternative way to induce proliferation, survival, motility, angiogenesis and invasive growth [24].

## 4. MET Pathway and SCLC

**Figure 2:**
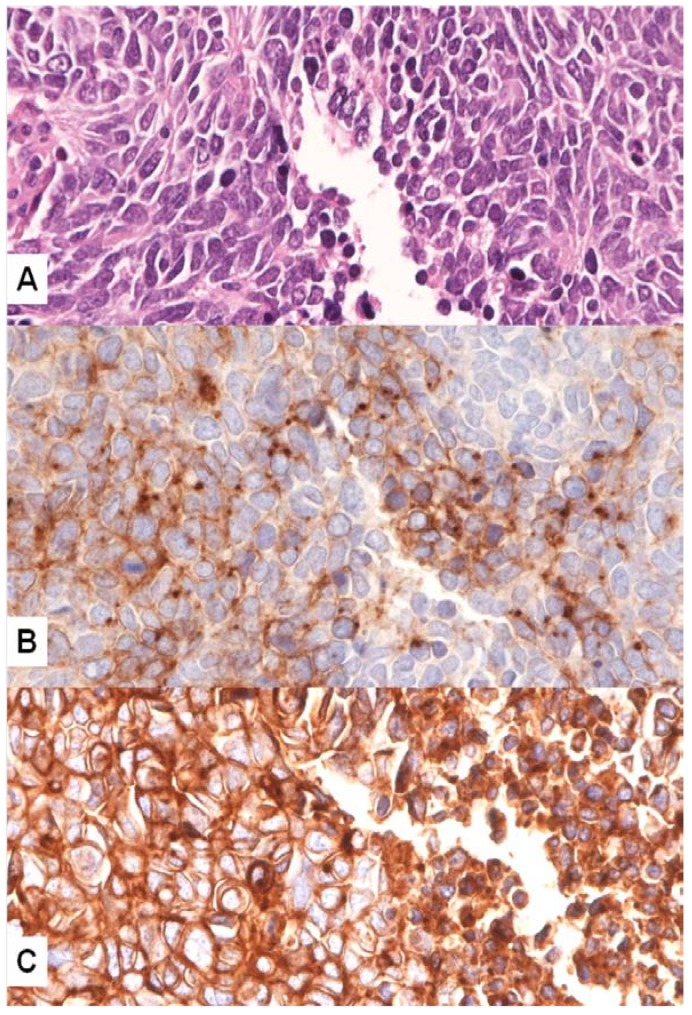
Consecutive/serial sections of a surgical biopsy of SCLC (**A**, hematoxylin-eosin stain, magnification ×400) with co-expression of SF/HGF in the tumor cell cytoplasm (**B**, immunohistochemistry, magnification ×400) and c-MET on the cell membrane (**C**, immunohistochemistry, magnification ×400).

Approximately 20 years ago, Rygaard *et al.* [57], evaluating a panel of 25 SCLC cell lines and xenografts, detected the expression of c-MET mRNA transcripts and c-MET protein in 88% of SCLC tumors; conversely, only two showed HGF mRNA levels and the co-expression of c-MET receptor/HGF was found in only one tumor. The authors concluded that this receptor/ligand system is frequently active in SCLC, possibly by a paracrine regulatory pathway. The same authors studied the effect of HGF on eight SCLC cell lines. A correlation between HGF stimulation and growth, scattering and invasiveness of those cell lines harboring c-MET receptor was shown [58]. Demonstration of co-expression of SF/HGF and c-MET by immunohistochemistry in the same tumor population seems to support that a subset of SCLC gains cell proliferation through an autocrine stimulatory mechanism (Figure 2A–C).

Later, another study demonstrated that the HGF/c-MET pathway is functional in SCLC. c-MET receptor expression was heterogeneous in various SCLC cell lines and highly phosphorylated when stimulated by its ligand; their interaction led to increased motility, cell migration and invasion, as well as higher production of reactive oxygen species (ROS). As a consequence of the aberrant c-MET/HGF signaling pathway, other adhesion proteins, such as paxillin, FAK, and PYK2, were active. By using an HSP90 inhibitor, all SCLC cell lines showed cell growth arrest and apoptosis, opening the way to a possible novel therapeutic approach [53].

Another study demonstrated that, in response to HGF, several specific c-MET tyrosine residues were phosphorylated, including Tyr1313, the binding site for PI3K. LY294002, a PI3K inhibitor, led to a significant reduction in cell growth, viability of SCLC, cell motility and migration. Therefore, PI3K had to be considered one of the c-MET downstream signaling pathways [59].

As previously mentioned, particularly in NSCLCs and upper gastrointestinal cancers, tumor progression can be sustained through an aberrant activation of the c-MET/HGF pathway due to c-MET/HGF overexpression, copy number gain/gene amplification or c-MET mutations. Conversely, according to available data for SCLC, the alterations able to induce an aberrant activation of c-MET are limited only to rare somatic mutations in the *c-MET* gene.

### MET Mutations in SCLC

Activating point mutations in the *MET* coding sequence have been reported as somatic and germline variants in many solid tumors, particularly in hereditary and sporadic papillary renal cell cancer [60], but also in lung tumors [61].

Ma *et al.* [18], sequencing *c-MET* gene, identified novel mutations in 3 out of 10 SCLC cell lines and in 4 out of 32 SCLC tumors. In cell lines, two different *c-MET* missense mutations (R988C and T1010I) in the juxtamembrane (JM) domain and one alternative transcript involving exon 10 were discovered for the first time. In SCLC tumors, two missense mutations were identified; one in the JM domain (T1010I) and the other in the Sema domain (E168D). In addition, 2 two-base-pair insertional mutations within the pre-JM intron 13 were detected. The presence of the JM domain mutations led to a constitutive protein tyrosine phosphorylation and resulted in altered morphology, adhesion and motility conferring an increased metastatic phenotype. Later, the same authors identified the E168D mutation in the Sema domain in one NSCLC tumor tissue (0.8%), the R988C mutation in the JM domain in one NSCLC cell line and both R988C and T1010I mutations in one tumor tissue (0.8%) [62].

In another study evaluating 44 SCLC tissue samples, none of the previous mutations was found, but two novel mutations were identified. There was a missense mutation in the JM domain (T995I) in a sample and a single amino-acid substitution in the Sema domain in two tumor tissues [19].

Voortman *et al.* reported a mutation rate of 25% in cell lines and 6.5% in clinical specimens [20]. Found mutations consisted of the previously reported R988C and T1010I mutations and a novel JM mutation (P996S) was detected in one SCLC specimen. On the contrary, in a retrospective analysis of 113 SCLC patients. mutation rate was 4.4%; interestingly, besides 4 mutations in the JM (2 R988C, 1 D990N and 1 D102Y), a novel activating missense mutation involving exon 17 (R1166Q) in the TK domain was identified. No correlation between MET and clinical-pathological features was detected in MET mutated SCLCs [21].

These results confirmed the low frequency of these mutations in SCLC (Figure 2), as well as in other lung tumors. It is important to note that the role of the MET mutation R988C is controversial in SCLC. Its role as driver mutation has not been clearly demonstrated; conversely, it seems to have a role as passenger mutation predisposing an individual toward cancer when combined with an oncogene that drives cellular proliferation [63].

## 5. Therapeutic Implications

There are different strategies developed to inhibit aberrant c-MET/HGF signaling pathway, including antibodies directed against HGF, c-MET receptor or decoy-MET, and small molecule directed against MET receptor tyrosine kinase domain or against downstream signaling modulators.

In SCLC, the proof of principle that MET could be an attractive therapeutic target was demonstrated by Ma *et al.* [64]. By using a phosphoproteomic approach in SCLC NCI-H69 cells, several downstream c-MET signaling transducers were identified, such as FAK, AKT, ERK1/2 and S6 kinase, involved in tumor invasion. Furthermore, the use of small interfering RNA (siRNA) and the selective c-MET inhibitor SU11274 correlated with the inhibition of c-MET phosphorylation, as well as that of the other transducers. Similar results were also reported by Wang *et al.* [65]. In their study, the authors showed that adenovirus-mediated siRNA could inhibit c-MET expression reducing SCLC cell growth and invasion both *in vitro* and in xenograft models, opening the way to a potential use of these technologies for the treatment of SCLC. Unfortunately, to date, no data from phase III trials are available on activity of MET inhibitors in SCLC.

### 5.1. MET Inhibitors and Their Potential Activity in Restoring Chemo-Sensitivity: Data from Preclinical Studies

In addition to its significance as predictor of poor outcome, in SCLC, the aberrant c-MET pathway plays a critical role as predictor of chemo-resistance.

Different studies showed that the presence of MET activation in *MET*-mutant SCLC cell lines was predictive of poor outcome and the use of PHA-665752, a selective c-MET inhibitor, inhibited colony formation and invasiveness [47].

In two recent studies, the same authors showed a correlation between HGF exposure/levels and epithelial-mesenchymal transition (EMT); this phenomenon resulted in a mesenchymal phenotype and in a worse outcome [66,67]. This process was accompanied by specific morphological and molecular changes, such as MET protein down-modulation, ERK1/2 activation and E-cadherin down-regulation. These changes were reversed or prevented *in vitro* by using MET inhibitors, such as crizotinib and PHA-665752 and translated *in vivo* in a significant decrease in tumor growth and local invasion. Furthermore, when crizotinib was added to etoposide, the highest inhibition rate both *in vitro* and *in vivo* was shown, suggesting that MET inhibition by crizotinib restored chemosensitivity [66].

Recently, another research group published the results of a study, confirming that a combination treatment might be a reasonable choice to treat a SCLC patient subgroup [68]. A significant positive correlation between the *MET* gene copy number and Top-I nuclear expression was detected, particularly in ED-SCLC tumors. Moreover, in SCLC H82 cells, a direct correlation between HGF stimulation and increased expression of phospho-MET and Top-I was demonstrated. This meant that Top-I activity was modulated by activation of the HGF/MET axis, suggesting a potential synergism of a dual block. Indeed, different cell lines were treated with SU11274 (a selective and small molecule MET tyrosine kinase inhibitor) and SN-38 (the active metabolite of irinotecan and a specific Topoisomerase-I inhibitor). Su11274 and SN-38 were highly active alone and even more in combination, particularly in H69 cells harboring R988C MET mutation; conversely, H82 cells (wild-type MET) were less sensitive to c-MET and Top-I inhibitors.

More recently, according to previous data, Ozasa *et al.* [69] addressed their attention to evaluate the significance of the deregulated HGF/MET pathway in acquired resistance to cytotoxic anticancer agents. By using quantitative real-time PCR and Western blot analysis, the authors reported significant differences in c-MET gene expression, c-MET, phospho-MET and HGF protein expression between specific human SCLC cell lines and the corresponding cells resistant to different cytotoxic agents. In drug-resistant cells, the use of SU11274 in combination with cytotoxic agents inhibited c-MET activation and altered resistance to the cytotoxic agents in a dose-dependent manner.

### 5.2. MET Inhibitors in Combination with Chemotherapy: Data from Clinical Studies

Data from phase 1/2 studies about chemotherapy in combination with AMG-102 (rilotumumab), a fully human IgG2 monoclonal antibody directed against HGF, have been presented. A Phase 1 dose-escalation study [70] of AMG-479 (ganitumab, a monoclonal antibody to IGF1R) or AMG-102 combined with platinum-based chemotherapy in untreated ED-SCLC showed an initial high incidence of venous thrombotic events (VTEs) in the AMG-102 cohorts and AMG-102 dose for phase II trial was set at 15 mg/kg and a carefully monitoring for TVE was recommended. In the subsequent 3-arm phase II trial [71] 185 untreated ED-SCLC patients were randomized 1:1:1 to receive rilotumumab (15 mg/kg day 1), ganitumab (18 mg/kg day 1) or placebo in combination with etoposide (100 mg/m^2^ IV days 1–3) plus cisplatin (75 mg/m^2^ day 1) or carboplatin (AUC 5 day 1) every three weeks up to 4–6 cycles followed by maintenance treatment with investigational drug. Primary end-point was overall survival (OS); secondary end-points were objective response rate (ORR), progression-free survival (PFS), pharmacokinetics and safety. Median OS in patients treated with rilotumumab plus chemotherapy (62) was 12.2 months and did not differ significantly than OS in the two other groups (10.7 and 10.8 months in ganitumab and placebo arms, respectively). There was also no difference between the three arms both in terms of PFS (5.4, 5.5 and 5.4 months, respectively) and ORR (68%, 63% and 59%, respectively). Two patients in the rilotumumab arm experienced a complete response. The treatment combinations were safe and tolerable without unexpected toxicities. Combined with chemotherapy, investigational drug exposure was comparable to that under monotherapy and did not affect the pharmacokinetics of chemotherapy. Survival analyses in biomarker and pharmacokinetic subgroups are ongoing.

## 6. Conclusions

SCLC remains one of the most aggressive tumors with a poor prognosis. The clinical outcome of these patients has reached its plateau with the standard treatment and new therapies are urgently required. In order to validate new effective drugs in SCLC, we first have to define the biological mechanisms underlying cancer promotion and progression, as was the case for NSCLC.

Thanks to the introduction of genomic and proteomic platforms, we will be increasingly able to understand the different molecular profilings of SCLC and to identify specific druggable targets, such as PTEN, FGFR1, SOX-2, PI3K, PARP and others.

To date, no target therapies are available in the treatment of SCLC. Paradoxically, several potential targets have been identified in SCLC and a plethora of promising biological agents are under investigation in clinical trials. Anti-angiogenic (bevacizumab, cediranib, sunitinib, pazopanib, vandetanib) drugs, mTOR (everolimus, temsirolimus), IGF1R (AMG479), HDAC (panobinostat, vorinostat), HSP90 (STA9090), Hedgehog (LY2940680), PARP (veliparib) inhibitors, anti-Bcl-2 compounds (oblimersen, AT-101), immunomodulating agents (ipilimumab) either alone or in combination with chemotherapy are in development for SCLC in different setting.

MET/HGF axis seems to be one of the most functional signaling pathways involved also in SCLC tumorigenesis and progression. Unlike NSCLCs and other tumors, in SCLC, the aberrant MET pathway is related only to activating mutations involving specific domains of c-MET receptor gene. These mutations are responsible for the constitutive activation of the MET signaling pathway and its downstream modulators, leading to an aggressive phenotype. Several data have demonstrated both *in vitro* and in xenografts that the inhibition of MET signaling cascades by using selective MET inhibitors correlates with cell growth arrest and apoptosis. Furthermore, the combination strategy (MET inhibitors plus cytotoxic agents) results in an additive antitumor effect and also restores chemo-sensitivity in *MET*-mutated resistant cells. Unfortunately, these results have as of yet not been confirmed in clinical practice. In fact, many MET inhibitors are currently being tested in several trials, but none of them has been certified for clinical use and no conclusions on their efficacy can be given. This discrepancy between preclinical and clinical assessments has to be ascribed both to the investigational drugs (pharmacokinetics and pharmacodynamics), and tumors’ (molecular targets) and patients’ selection (pharmacogenomics, circulating biomarkers).

In conclusion, MET inhibitors may also be a promising arm in the battle against SCLC, but more questions remain unsolved. We have thus far been unable to accurately identify the tumors and patient subgroups that can benefit from MET inhibition, as well as the best treatment and combination strategies and the most appropriate setting. Finally, we need to detect new circulating biomarkers to predict a better outcome from MET inhibitors. Only in finding the answers to these questions will we be able to improve the treatment strategy of SCLC and, ultimately, the outcome of these patients.
